# Can’t RIDD off viruses

**DOI:** 10.3389/fmicb.2014.00292

**Published:** 2014-06-18

**Authors:** Sankar Bhattacharyya

**Affiliations:** Vaccine and Infectious Disease Research Centre, Translational Health Science and Technology InstituteGurgaon, India

**Keywords:** unfolded protein response, UPR, RNaseL, OAS, IRE1, Xbp1, RIDD pathway

## Abstract

The mammalian genome has evolved to encode a battery of mechanisms, to mitigate a progression in the life cycle of an invasive viral pathogen. Although apparently disadvantaged by their dependence on the host biosynthetic processes, an immensely faster rate of evolution provides viruses with an edge in this conflict. In this review, I have discussed the potential anti-virus activity of inositol-requiring enzyme 1 (IRE1), a well characterized effector of the cellular homeostatic response to an overloading of the endoplasmic reticulum (ER) protein-folding capacity. IRE1, an ER-membrane-resident ribonuclease (RNase), upon activation catalyses regulated cleavage of select protein-coding and non-coding host RNAs, using an RNase domain which is homologous to that of the known anti-viral effector RNaseL. The latter operates as part of the Oligoadenylate synthetase OAS/RNaseL system of anti-viral defense mechanism. Protein-coding RNA substrates are differentially treated by the IRE1 RNase to either augment, through cytoplasmic splicing of an intron in the *Xbp1* transcript, or suppress gene expression. This referred suppression of gene expression is mediated through degradative cleavage of a select cohort of cellular RNA transcripts, initiating the regulated IRE1-dependent decay (RIDD) pathway. The review first discusses the anti-viral mechanism of the OAS/RNaseL system and evasion tactics employed by different viruses. This is followed by a review of the RIDD pathway and its potential effect on the stability of viral RNAs. I conclude with a comparison of the enzymatic activity of the two RNases followed by deliberations on the physiological consequences of their activation.

## INTRODUCTION

**FIGURE 1:**
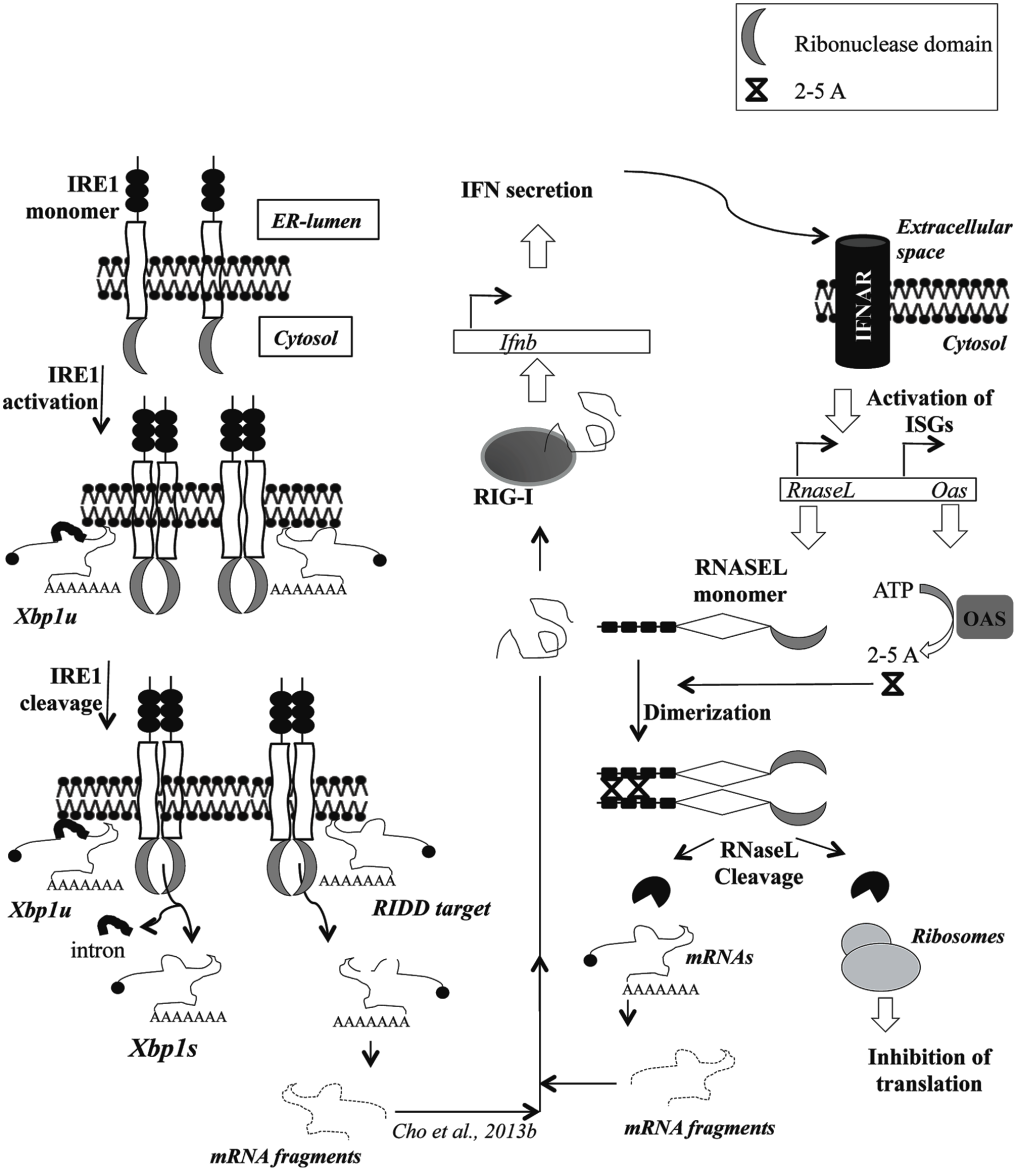
**Schematic representation of the ribonuclease activity of IRE1 and RNaseL showing cross-talk between the paths catalysed by the enzymes.** The figure shows activation of RNase activity following dimerization triggered by either accumulation of unfolded proteins in the ER-lumen or synthesis of 2–5A by the enzyme OAS, respectively, for IRE1 and RNaseL. The cleavage of *Xbp1u* by IRE1 releases an intron thus generating *Xbp1s*. The IRE1 targets in RIDD pathway or all RNaseL substrates are shown to undergo degradative cleavage. The cleavage products generated through degradation of the respective substrate is shown to potentially interact with RIG-I thereby leading to Interferon secretion and trans-activation of *Oas* genes through Interferon signaling. Abbreviations: RIG-I = retinoic acid inducible gene-I, Ifnb = interferon beta gene loci, IFN = interferons, ISG = interferon-sensitive genes, 2–5A = 2′–5′ oligoadenylates.

Establishment prove to be inhibitory for the viral life cycle in a direct or an indirect manner. The direct mechanism involves expression of multiple anti-viral genes that have evolved to recognize, react, and thereby rid the infected host of the viral nucleic acid (Zhou et al., 1997; Thompson et al., 2011). On the other hand the pathways, e.g., those that culminate in initiating an apoptotic death for the host cell, indirectly serve to limit the spread of virus (Roulston et al., 1999). A major difference between these two mechanisms is that while the former response is transmissible to neighboring uninfected cells through interferon (IFN) signaling, the latter is observed mostly in *cis*. Recent reports, however, have demonstrated transmission of an apoptotic signal between cells that are in contact through gap junctions, although such a signaling from an virus infected host cell to an uninfected one is not known yet (Cusato et al., 2003; Udawatte and Ripps, 2005; Kameritsch et al., 2013). Successful viral pathogens, through a process of active selection, have evolved to replicate and simultaneously evade or block either of these host responses. The viral nucleic acids which could be the genome (positive-sense single-stranded RNA virus) or RNA derived from transcription of the genome [negative-stranded single-sense RNA or double-stranded RNA (dsRNA) or DNA virus], offer critical targets for both detection and eradication. The viral nucleic acid targeting armaments in the host arsenal include those that recognize the associated molecular patterns like toll-like receptors (TLRs), DDX58 (or RIG-1), IFIH1 (or MDA5), IFIT proteins [IFN-stimulated genes (ISG)56 and ISF54], etc. (Aoshi et al., 2011; Bowzard et al., 2011; Jensen and Thomsen, 2012). This is followed by IFN signaling and expression or activation of factors that target the inducer for degradation or modification like OAS/ribonuclease L (RNaseL) system, APOBEC3, MCPIP1, the ZC3HAV1/exosome system and RNAi pathways (Gao et al., 2002; Sheehy et al., 2002; Guo et al., 2007; Daffis et al., 2010; Sidahmed and Wilkie, 2010; Schmidt et al., 2012; Cho et al., 2013a; Lin et al., 2013). In this review we focus on two proteins containing homologous RNase domains, RNaseL with a known direct antiviral function and Inositol-requiring enzyme 1 (IRE1 or ERN1) which has an RNaseL-like RNase domain with a known role in homeostatic response to unfolded proteins in the endoplasmic reticulum (ER) and a potential to function as an antiviral (**Figure 1**; Tirasophon et al., 2000).

## DEGRADATION OF VIRAL RNA BY RNaseL AND VIRAL EVASION

In mammalian cells the tell-tale signs of RNA virus infection, like the presence of cytosolic RNA having 5′-ppp or extensive (>30 bp) dsRNA segments are detected by dedicated pathogen associated molecular pattern receptors (PAMPs) or pattern recognition receptors (PRRs) in the host cell, like RIG-1, MDA5, and the IFIT family of proteins (Aoshi et al., 2011; Bowzard et al., 2011; Vabret and Blander, 2013). The transduction of a signal of this recognition results in the expression of IFN genes the products of which upon secretion outside the cell bind to cognate receptors, initiating further downstream signaling (**Figure 1**; Randall and Goodbourn, 2008). The genes that are regulated as a result of IFN signaling are termed as IFN-stimulated or IFN-regulated genes (ISGs or IRGs; Sen and Sarkar, 2007; Schoggins and Rice, 2011). Oligoadenylate synthetase or OAS genes are canonical ISGs that convert ATP into 2′–5′ linked oligoadenylates (2–5A) by an unique enzymatic mechanism (**Figure 1**; Hartmann et al., 2003). Further, they are RNA-binding proteins that function like PRRs, in a way that the 2–5A synthesizing activity needs to be induced through an interaction with dsRNA (Minks et al., 1979; Hartmann et al., 2003). In a host cell infected by an RNA virus, such dsRNA is present in the form of replication-intermediates (RI), which are synthesized by the virus-encoded RNA-dependent RNA polymerases (RdRp) and subsequently used by the same enzyme to synthesize more genomic RNA, through asymmetric transcription (Weber et al., 2006). However, the replications complexes (RCs) harboring these RI molecules are found secluded inside host-membrane derived vesicles, at least in positive-strand RNA viruses, a group which contains many human pathogens (Uchil and Satchidanandam, 2003; Denison, 2008). Reports from different groups suggest OAS proteins to be distributed both in the cytoplasm as well as in membrane-associated fractions, perhaps indicating an evolution of the host anti-viral methodologies towards detection of the membrane-associated viral dsRNAs (Marie et al., 1990; Lin et al., 2009). DNA viruses on the other hand, produce dsRNA by annealing of RNA derived from transcription of both strands in the same viral genomic loci, which are probably detected by the cytoplasmic pool of OAS proteins (Jacobs and Langland, 1996; Weber et al., 2006). Post-activation the OAS enzymes synthesize 2–5A molecules in a non-processive reaction producing oligomers which, although potentially ranging in size from dimeric to multimeric, are functionally active only in a trimeric or tetrameric form (Dong et al., 1994; Sarkar et al., 1999; Silverman, 2007). These small ligands, which bear phosphate groups (1–3) at the 5′ end and hydroxyl groups at the 2′ and 3′ positions, serve as co-factor which can specifically interact with and thereby allosterically activate, existing RNaseL molecules (Knight et al., 1980; Zhou et al., 1997, 2005; Sarkar et al., 1999). As part of a physiological control system these 2–5A oligomers are quite unstable in that they are highly susceptible to degradation by cellular 5′-phosphatases and PDE12 (2′-phosphodiesterase; Silverman et al., 1981; Johnston and Hearl, 1987; Kubota et al., 2004; Schmidt et al., 2012). Viral strategies to evade or overcome this host defense mechanism ranges from preventing IFN signaling which would hinder the induction of OAS expression or thwarting activation of expressed OAS proteins by either shielding the viral dsRNA from interacting with it or modulating the host pathway to synthesize inactive 2–5A derivatives (Cayley et al., 1984; Hersh et al., 1984; Rice et al., 1985; Maitra et al., 1994; Beattie et al., 1995; Rivas et al., 1998; Child et al., 2004; Min and Krug, 2006; Sanchez and Mohr, 2007; Sorgeloos et al., 2013). Shielding of viral RNA from interacting with OAS is possible through enclosure of dsRNA replication intermediates in membrane enclosed compartments as observed in many flaviviruses (Ahlquist, 2006; Miller and Krijnse-Locker, 2008; Miorin et al., 2013).

**FIGURE 2:**
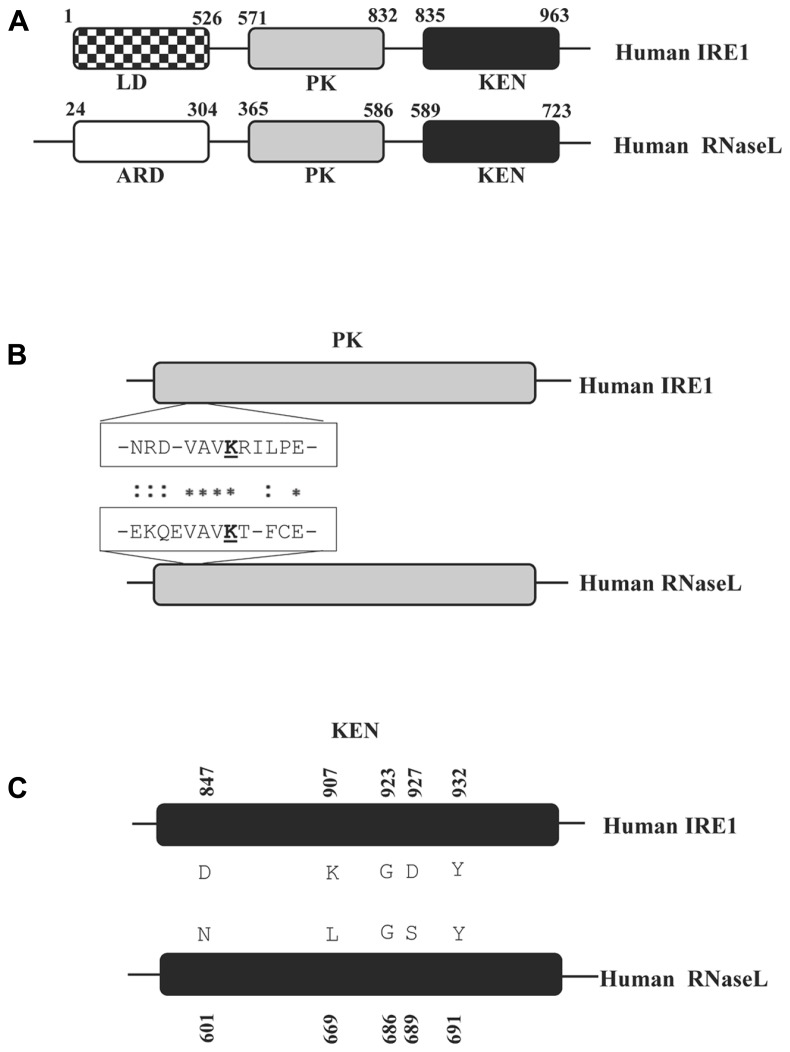
**Schematic representation of distinct protein domains in human RNaseL and IRE1. (A)** The domains homologous between RNaseL and IRE1 are shaded identically. The domain name abbreviations denote the following: ARD = ankyrin repeat domain; LD = luminal domain; PK = protein kinase domain; KEN = kinase extension nuclease domain. The amino acid positions bordering each domain are numbered. The schematic drawings are not according to scale. **(B)** ClustalW alignment of primary sequence from a segment of the PK domain indicating amino acid residues which are important for interacting with nucleotide cofactors. The conserved lysine residues, critical for this interaction (K599 for IRE1 and K392 in RNaseL) are underlined. **(C)** Alignment of the KEN domains in RNaseL and IRE1. The amino acids highlighted and numbered in IRE1 are critical for the IRE1 RNase activity (Tirasophon et al., 2000).

RNaseL is a 741 amino acid protein containing three predominantly structured region, an N-terminal ankyrin repeat domain (ARD), a middle catalytically inactive pseudo-kinase (PK) and a C-terminal RNase domain (**Figure 2A**; Hassel et al., 1993; Zhou et al., 1993). The activity of the RNase domain is negatively regulated by the ARD, which is relieved upon binding of 2–5A molecules to ankyrin repeats 2 and 4 followed by a conformational alteration (**Figure 1**; Hassel et al., 1993; Tanaka et al., 2004; Nakanishi et al., 2005). In support of this contention, deletion of the ARD has been demonstrated to produce constitutively active RNaseL, although with dramatically lower RNase activity (Dong and Silverman, 1997). However, recent reports suggest that while 2–5A links the ankyrin repeats from adjacent molecules leading to formation of dimer and higher order structures, at sufficiently high *in vitro* concentrations, RNaseL could oligomerize even in the absence of 2–5A (Han et al., 2012). Nonetheless, *in vivo* the RNaseL nuclease activity still seems to be under the sole regulation of 2–5A (Al-Saif and Khabar, 2012). In order to exploit this dependence, multiple viruses like mouse hepatitis virus (MHV) and rotavirus group A (RVA) have evolved to encode phosphodiesterases capable of hydrolysing the 2′–5′ linkages in 2–5A and thereby attenuate the RNaseL cleavage activity (Zhao et al., 2012; Zhang et al., 2013). In addition to 5′-phosphatases and 2′-phosphodiesterases to reduce the endogenous 2–5A levels, mammalian genomes encode post-transcriptional and post-translation inhibitors of RNaseL activity in the form of microRNA-29 and the protein ABCE1 (RNaseL inhibitor or RLI), respectively (Bisbal et al., 1995; Lee et al., 2013). Direct inhibition of RNaseL function is also observed upon infection by Picornaviruses through, either inducing the expression of ABCE1 or exercising a unique inhibitory property of a segment of the viral RNA (Martinand et al., 1998, 1999; Townsend et al., 2008; Sorgeloos et al., 2013).

Once activated by 2–5A, RNaseL can degrade single-stranded RNA irrespective of its origin (virus or host) although there seems to exist a bias towards cleavage of viral RNA (Wreschner et al., 1981a; Silverman et al., 1983; Li et al., 1998). RNA sequences that are predominantly cleaved by RNaseL are U-rich with the cleavage points being typically at the 3′ end of UA or UG or UU di-nucleotides, leaving a 5′-OH and a 3′-monophosphate in the cleavage product (Floyd-Smith et al., 1981; Wreschner et al., 1981b). A recent report shows a more general consensus of 5′-UNN-3′ with the cleavage point between the second and the third nucleotide (Han et al., 2014). Cellular targets of RNaseL include both ribosomal RNA (rRNA) and mRNAs, the latter predominantly representing genes involved in protein biosynthesis (Wreschner et al., 1981a; Al-Ahmadi et al., 2009; Andersen et al., 2009). Additionally, RNaseL activity can also degrade specific ISG mRNA transcripts and thereby attenuate the effect of IFN signaling (Li et al., 2000). Probably an evolution towards insulating gene expression from RNaseL activity is observed in the coding region of mammalian genes where the UU/UA dinucleotide frequency is rarer (Bisbal et al., 2000; Khabar et al., 2003; Al-Saif and Khabar, 2012). Perhaps not surprisingly, with a much faster rate of evolution, similar observations have been made with respect to evasion of RNaseL mediated degradation by viral RNAs too (Han and Barton, 2002; Washenberger et al., 2007). Moreover, nucleoside modifications in host mRNAs, rarely observed in viral RNAs, have also been shown to confer protection from RNaseL (Anderson et al., 2011). In addition to directly targeting viral RNA, the reduction in functional ribosomes and ribosomal protein mRNA affects viral protein synthesis and replication in an indirect manner. Probably, as a reflection of these effects on cellular RNAs, RNaseL is implicated as one of the factors determining the anti-proliferative effect of IFN activity (Hassel et al., 1993). The anti-viral activity of RNaseL extends beyond direct cleavage of viral RNA, through stimulation of RIG-I by the cleavage product (Malathi et al., 2005, 2007, 2010). A global effect of RNaseL is observed in the form of autophagy induced through c-jun N-terminal kinase (JNK) signaling and apoptosis, probably as a consequence of rRNA cleavage (Li et al., 2004; Chakrabarti et al., 2012; Siddiqui and Malathi, 2012). RNaseL has also been demonstrated to play a role in apoptotic cell death initiated by pharmacological agents extending the physiological role of this pathway beyond the boundary of being only an anti-viral mechanism (Castelli et al., 1997, 1998).

## IRE1 AND THE RIDD PATHWAY

The ER serves as a conduit for maturation of cellular proteins which are either secreted or destined to be associated with a membrane for its function. An exclusive microenvironment (high Calcium ion and unique ratio of reduced to oxidized glutathione) along with a battery of ER-lumen resident enzymes (foldases, chaperones, and lectins) catalyse/mediate the necessary folding, disulfide-bond formation, and glycosylation reactions (Schroder and Kaufman, 2005). A perturbation of the folding capacity, due to either physiological disturbances or virus infection, can lead to an accumulation of unfolded proteins in the ER lumen, which signals an unfolded protein response (UPR). UPR encompasses a networked transcriptional and translational gene-expression program, initiated by three ER-membrane resident sensors namely IRE1 or ERN1, PKR-like ER Kinase (PERK or EIF2AK3) and activating transcription factor 6 (ATF6; Hetz, 2012). IRE1 is a type I single-pass trans-membrane protein in which, similar to what is observed with RNaseL, the N-terminal resident in the ER lumen serves as sensor and the cytosolic C-terminal as the effector (**Figure 1**; Chen and Brandizzi, 2013). The IRE1 coding gene is present in genomes ranging from yeast to mammals and in the latter is ubiquitously expressed in all tissues (Tirasophon et al., 1998). Signal transduction by stimulated IRE1 initiates multiple gene regulatory pathways with either pro-survival or pro-apoptotic consequences (Kaufman, 1999). During homeostasis or unstressed conditions the sensor molecules are monomeric, a state maintained co-operatively by the “ absence” of unfolded proteins and the “presence” of HSPA5 (GRP78 or Bip, an ER-resident chaperone) molecules bound to a membrane-proximal disordered segment of the protein in the ER-lumen-resident N-terminus (Credle et al., 2005). Accumulated unfolded proteins in the lumen triggers coupling of this domain from adjacent sensor molecules through a combination of (a) titration of the bound HSPA5 chaperone molecules and (b) direct tethering by malfolded protein molecules (Shamu and Walter, 1996; Credle et al., 2005; Aragon et al., 2009; Korennykh et al., 2009). Abutting of the luminal domains juxtapose the cytosolic C-terminal segments, leading to an aggregation of the IRE1 molecules into distinct ER-membrane foci (Kimata et al., 2007; Li et al., 2010). The C-terminal segment has a serine/threonine kinase domain and a RNase domain homologous to that of RNaseL (**Figure 1**; Tirasophon et al., 1998, 2000). A *trans*-autophosphorylation by the kinase domain allosterically activates the RNase domain (Tirasophon et al., 2000; Lee et al., 2008; Korennykh et al., 2009). In fact, exogenous over-expression of IRE1 in mammalian cells lead to activation suggesting that, under homeostatic conditions, the non-juxtaposition of cytosolic domains maintains an inactive IRE1 (Tirasophon et al., 1998). Once activated, IRE1 performs cleavage of a variety of RNA substrates mediated by its RNase domain, in addition to phosphorylating and thereby activating JNK (Cox and Walter, 1996; Urano et al., 2000). Depending on the RNA substrate, the cleavage catalyzed by IRE1 RNase produces differential consequence. Although scission of the *Xbp1* mRNA transcript at two internal positions is followed by splicing of the internal segment through ligation of the terminal cleavage products, that in all other known IRE1 target RNA is followed by degradation (**Figure 1**; Sidrauski and Walter, 1997; Calfon et al., 2002). The latter mode of negative regulation of gene expression is termed as the regulated IRE1-dependent decay (RIDD) pathway (Hollien and Weissman, 2006; Oikawa et al., 2007; Iqbal et al., 2008; Lipson et al., 2008). Gene transcripts regulated by RIDD pathway includes that from IRE1 (i.e., self-transcripts), probably in a negative feedback loop mechanism (Tirasophon et al., 2000). In addition to protein coding RNA, RIDD pathway down-regulates the level of a host of microRNA precursors (pre-miRNAs) and can potentially cleave in the anti-codon loop of tRNA^Phe^ (Korennykh et al., 2011; Upton et al., 2012).

The IRE1 RNase domain cleaves the *Xbp1u* (u for unspliced) mRNA transcript at two precise internal positions within the open reading frame (ORF) generating three segments, the terminal two of which are ligated by a tRNA ligase in yeast and by an unknown ligase in mammalian cells, to produce the *Xbp1s* (s for spliced) mRNA transcript (**Figure 1**; Yoshida et al., 2001). The *Xbp1s* thus generated has a longer ORF, which is created by a frame-shift in the coding sequence downstream of the splice site (Cox and Walter, 1996; Calfon et al., 2002). A similar dual endonucleolytic cleavage is also observed to initiate the XRN1 and Ski2-3-8 dependent degradation of transcripts in the RIDD degradation pathway (Hollien and Weissman, 2006). The RIDD target transcript genes are predominantly those that encode membrane-associated or secretory proteins and which are not necessary for ER protein-folding reactions (Hollien and Weissman, 2006). The cleavage of *Xbp1* and the RIDD-target transcripts constitute homeostatic or pro-survival response by IRE1 since XBP1S *trans*-activates genes encoding multiple chaperones (to fold unfolded proteins) and the ERAD pathway genes (to degrade terminally misfolded proteins) whereas RIDD reduces flux of polypeptides entering the ER lumen (Lee et al., 2003; Hollien and Weissman, 2006). On the other hand, cleavage of pre-miRNA transcripts which are processed in the cell to generate CASPASE-2 mRNA (*Casp2*) controlling miRNAs, constitutes the pro-apoptotic function of IRE1 (Upton et al., 2012). Another pro-apoptotic signal from IRE1 emanates from signaling through phosphorylation of JNK1 (Urano et al., 2000). Although in the initial phase RIDD activity does not cleave mRNAs encoding essential ER proteins, at later stages of chronic UPR such transcripts are rendered susceptible to degradation promoting apoptosis induction (Han et al., 2009; Bhattacharyya et al., 2014).

Infection of mammalian cells by a multitude of viruses induce an UPR which is sometimes characterized by suppression of signaling by one or more of the three sensor(s; Su et al., 2002; Tardif et al., 2002; He, 2006; Yu et al., 2006, 2013; Medigeshi et al., 2007; Zhang et al., 2010; Merquiol et al., 2011). Among these at least two viruses from diverse families, HCMV (a DNA virus) and hepatitis C virus (a hepacivirus), interfere with IRE1 signaling by different mechanism (Tardif et al., 2004; Stahl et al., 2013). An observed inhibition of any cellular function by a virus infection could suggest a potential anti-virus function for it, which the virus has evolved to evade through blocking some critical step(s). In both the cases mentioned above, stability of the viral proteins seems to be affected by ERAD-mediated degradation, although other potential anti-viral effect of IRE1 activation are not clear yet (Isler et al., 2005; Saeed et al., 2011). Interestingly, host mRNA fragments produced following IRE1 activation during bacterial infection, has been shown to activate RIG-I signaling (**Figure 1**; Cho et al., 2013b). Theoretically, other functions of IRE1 can also have anti-viral effect necessitating its inhibition for uninhibited viral replication. It is, however, still not clear whether IRE1 is able to cleave any viral RNA (or mRNA) in a manner similar to that of other RIDD targets (**Figure 1**). The possibilities of such a direct anti-viral function are encouraged by the fact that all these viruses encode at least one protein which, as part of its maturation process, requires glycosylation and disulfide-bond formation. Such a necessity would entail translation of the mRNA encoding such a protein, which in case of positive-sense single-stranded RNA viruses would mean the genome, in association with the ER-membrane (**Figure 1**; Lerner et al., 2003). Additionally for many RNA viruses, replication complexes are housed in ER-derived vesicular structures (Denison, 2008; den Boon et al., 2010). Considering the proximity of IRE1 and these virus-derived RNAs it is tempting to speculate that probably at some point of time in the viral life cycle one or more virus-associated RNA would be susceptible to cleavage by IRE1. However, studies with at least two viruses have shown that instead of increasing viral titre, inhibiting the RNase activity of activated IRE1 has an opposite effect (Hassan et al., 2012; Bhattacharyya et al., 2014). This implies potential benefits of IRE1 activation through one or more of the following, (a) expression of chaperones or other pro-viral molecules downstream of XBP1S-upregulation or JNK-activation, (b) cleavage of potential anti-viral gene mRNA transcripts by RIDD activity. However, the mode of protection for the viral RNA from RIDD activity is still not clear. It is possible that the viral proteins create a subdomain within the ER membrane, which through some mechanism excludes IRE1 from diffusing near the genomic RNA, thereby protecting the replication complexes (Denison, 2008). It is therefore probably not surprising that single-stranded plus-sense RNA viruses encode a polyprotein, which produces replication complexes in *cis*, promoting formation of such subdomains (Egger et al., 2000). The fact that IRE1 forms bulky oligomers of higher order probably aggravates such an exclusion of the activated sensor molecules from vicinity of the viral replication complexes. The UPR signaling eventually attenuate during chronic ER-stress and since that is what a virus-induced UPR mimics, probably the viral RNA needs protection only during the initial phase of UPR activation (Lin et al., 2007). Since the choice of RIDD target seems to be grossly driven towards mRNAs that encode ER-transitory but are not ER-essential proteins, it is also possible that one or more viral protein have evolved to mimic a host protein the transcript of which is RIDD-resistant (Hollien and Weissman, 2006). Most of the RIDD target mRNA are observed to be ER-membrane associated, the proximity to IRE1 facilitating association and cleavage (**Figure 1**; Hollien and Weissman, 2006). Although ER-association for an mRNA is possible without the mediation of ribosomes, Gaddam and co-workers reported that continued association with polysomes for a membrane-bound mRNA can confer protection from IRE1 cleavage (Cui et al., 2012; Gaddam et al., 2013). This would suggest important implications for the observed refractory nature of Japanese encephalitis virus (JEV) and influenza virus RNA to RIDD cleavage (Hassan et al., 2012; Bhattacharyya et al., 2014). In contrast to Influenza virus, flaviviruses (which include JEV) do not suppress host protein synthesis implying the absence of a global inhibition on translation as would be expected during UPR (Clyde et al., 2006; Edgil et al., 2006). Therefore, a continued translation of viral RNA in spite of UPR activation can in principle confer protection from the pattern of RNA cleavage observed in the RIDD pathway.

## COMPARISON OF IRE1 AND RNaseL

IRE1 and RNaseL, in addition to biochemical similarities in protein kinase domain and structural similarities in their RNase domain, share the functional consequences of their activation in initiating cellular apoptosis through JNK signaling (**Table 1** and **Figure 2**; Liu and Lin, 2005; Dhanasekaran and Reddy, 2008). Though initial discoveries were made in the context of homeostatic and anti-viral role for the former and latter, differences between the pathways are narrowed by further advances in research. In the same vein, while inhibition of IRE1 signaling in virus infected cells indicates a potential anti-viral role, association of RNaseL mutations with generation of prostate cancer extends the ambit of influence of this anti-viral effector to more non-infectious physiological disorders (Silverman, 2003). Biochemically, the similarity in their RNase domains does not extend to the choice of either substrates or cleavage point, which are downstream of UU or UA in RNaseL and downstream of G (predominantly) for IRE1 (**Figure 2C**; Yoshida et al., 2001; Hollien and Weissman, 2006; Upton et al., 2012). Further, while RNaseL cleaves pre-dominantly in single-stranded region, IRE1 seems to cleave equally well in single- and double-stranded region (Upton et al., 2012). However, a recent report suggested a consensus cleavage site with the sequence UN/N, in RNaseL targets and in those mRNAs that are cleaved by IRE1 as part of the RIDD pathway (Han et al., 2014). Access to potential cleavage substrate for RNaseL is conjectured to be facilitated through its association with polyribosomes, while no such association is known for IRE1 (Salehzada et al., 1991). Possibilities exist that IRE1 would have preferential distribution in the rough ER which, upon activation, would give it ready access to mRNAs for initiating the RIDD pathway.

**Table 1:** A comparison of the structural and biochemical properties of RNaseL and IRE1, showing similarities and differences.

	Similarities	
	RNaseL	IRE1
Inactive state	Monomeric
Active state	Oligomeric
Factor driving oligomerization	Catenation of by 2–5A bound to ankyrin repeats of multiple monomers	Titration of HSPA5 bound to luminal domain and catenation of the same from multiple monomers by unfolded proteins
Activation upon exogenous overexpression	Yes (demonstrated *in vitro* for RNaseL)
Position of ligand–receptor and RNase domain	N- and C-terminal, respectively
Ribonuclease domain	KEN or kinase-extension homology domain
Role of PK domain in activating RNase	Nucleotide binding, even in absence of hydrolysis, to conserved residue in protein-kinase like domain is necessary for RNase activity (Tirasophon et al., 1998; Dong and Silverman, 1999; Papa et al., 2003; Lin et al., 2007)	
Nature of RNase substrates	Both 28S rRNA and mRNAs	IRE1β can cleave both 28S rRNA and mRNA while IRE1α substrates include only mRNAs (Iwawaki et al., 2001)
**Dissimilarities**
Autophosphorylation	No	Yes
Cleavage substrates	Beside 28S rRNA, predominantly cleaves mRNAs encoding ribosomal proteins (Andersen et al., 2009)	*Xbp1u* and other mRNAs in addition to microRNA precursors which are targeted as part of the RIDD pathway
Selection of cleavage site	Cleaved between 2nd and 3rd nucleotide positions of UN/N sites (Han et al., 2014)	RNA sequence with the consensus of 5′-CUGCAG-3′ in association with a stem-loop (SL) structure essential for recognition of *Xbp1u* and other mRNAs (Oikawa et al., 2010)

In the context of a virus infection, the pathway leading from both these proteins have the potential to lead to cell death. Notwithstanding the fact that this might be an efficient way of virus clearance, it also portends pathological outcomes for the infected organism. Future research would probably lead to design of drugs targeting these proteins based on the structural homology of their effector domains, regulating the pathological denouement of their activation without compromising their anti-viral or potential anti-viral functions.

## Conflict of Interest Statement

The author declares that the research was conducted in the absence of any commercial or financial relationships that could be construed as a potential conflict of interest.
